# The Swedish fracture register: 103,000 fractures registered

**DOI:** 10.1186/s12891-015-0795-8

**Published:** 2015-11-06

**Authors:** David Wennergren, Carl Ekholm, Anna Sandelin, Michael Möller

**Affiliations:** Department of Orthopedics, Sahlgrenska University Hospital Gothenburg/ Mölndal, SE-431 80 Mölndal, Sweden; Center of Registers, Western Healthcare Region, SE-413 45 Gothenburg, Sweden

**Keywords:** Fracture, Register, Epidemiology, Patient-reported outcome measure

## Abstract

**Background:**

Although fractures consume large social and financial resources, little is known about their actual numbers, treatment methods or outcomes. The scarcity of data calls for a high-quality, population-based register. No previous registers have prospectively collected data and patient-reported outcome measures (PROMs) on fractures of all types. The Swedish Fracture Register was recently created to fill this gap in knowledge. Its purpose is to provide information on fractures of all types, whether treated by surgery or otherwise. The aim of this article is to describe how the register was developed and its current use.

**Description:**

The Swedish Fracture Register was developed during a 4-year period, 2007–2010. Data collection started in 2011. The register currently collects data on all extremity, pelvic and spine fractures in adults who have been diagnosed or treated at the affiliated departments. Data entry is fully web based, including date, cause of injury, classification and treatment. It is performed by the attending physician. Patients fill out PROMs – EQ-5D-3L and the Short Musculoskeletal Function Assessment (SMFA) – relating to health status and level of functioning before the fracture and one year later. Surgeon-reported outcome measures are registered as reoperation rates. The Swedish Fracture Register is now functioning effectively and is used in clinical routine. From January 2011 to September 2015, more than 103,000 fractures have been entered at 26 Swedish orthopedic departments.

**Conclusions:**

The Swedish Fracture Register is already a well-functioning, population-based fracture register that covers fractures of all types, regardless of treatment, and collects both surgeon- and patient-reported outcome measures. In the future the Swedish Fracture Register will be able to present both results of fracture treatment and valuable epidemiological data.

## Background

Although fracture care consumes large social and financial resources, little is known about outcomes, methods or the actual number of fractures treated each year. The same is largely true of reoperation rates or patient-reported outcome measures (PROM). Data collection usually takes place indirectly from public health registers. The main limitations of the present systems include the lack of accurate codes in the medical records, as well as the inability to indicate the affected side or the presence of bilateral fractures. According to the Swedish Patient Register, an estimated 140,000 fractures are treated in Sweden each year. However, national data based on classifications and assessments by orthopedic surgeons are scarce.

Ever since the Swedish Knee Arthroplasty Register was launched in 1975 and the Swedish Hip Arthroplasty Register in 1979, quality registers have had a major impact on orthopedic treatment [1–3]. Quality registers enable scientific assessments in areas for which randomized, controlled trials are not always possible [4]. When the absolute risk of complications is low, quality registers are able to detect crucial differences, while randomized, controlled trials may not include enough patients to do this [5, 6]. Patient-reported outcomes are important and have been prospectively collected and evaluated for several years in the Swedish Hip Arthroplasty Register [7]. In value-based health care, patient-reported outcome is one of the cornerstones.

Due to unique Swedish personal identity numbers, patients’ data can be entered in registers and monitored over time. The personal identity numbers make it possible to follow the patients, even when they are treated by different providers.

There is a widely recognized need for population-based register data in order to determine resource allocation, promote better outcomes and develop evidence-based trauma orthopedics. National registers that focus on specific fractures and regional registers that specialize in surgical treatment have been established [8–10]. The Norwegian Hip Fracture Register has provided valuable knowledge on the treatment and outcome of hip fractures [8]. The Fracture and Dislocation Registry (FDR) at Stavanger University Hospital and the Danish Fracture Database are both interesting examples of important work to create fracture registers [9, 10]. However, they collect data on surgically treated fractures only and do not collect PROMs. Alongside this a large epidemiological fracture survey with the classification of more than 100,000 fractures has been presented by a Chinese centre, but neither treatment nor results are included [11]. Despite these efforts, until now, there have been no national registers that prospectively collect data on fractures of all types, regardless of location and type of treatment, as well as patient-reported outcome measures. The creation of the SFR was based on the hypothesis that it is possible to create a population-based fracture register that covers fractures of all types, regardless of treatment, and collects both surgeon- and patient-reported outcome measures. The hypothesis is also that a national fracture register can collect more detailed information in terms of the fracture type and its treatment than official health statistics can provide. The aim of the Swedish Fracture Register (SFR) is to provide population-based data on the outcomes of fracture treatment including reoperation rates and PROM and to serve as a basis for improving the health-care system. The aim of this article is to review the development, implementation and current use of the register. Data from the register will be presented in forthcoming articles.

## Construction and content

### How the SFR was developed

The SFR was created by orthopedic surgeons to fill the gap in knowledge relating to the treatment of fractures. The process of defining the variables to be included started in 2007. The number of variables in any register that aims to include all fractures has to be limited. The main outcome measure, as in most other registers studying surgical interventions, was chosen to be reoperation rates. Registration of the reoperations subdivided by reasons for the reoperation will cover most complications as deep infection, mal-union, non-union etc. Each chosen variable must add valuable information. Otherwise it has to be discarded because of the risk of non-compliance if the process of data entry becomes too time-consuming. The structure of the register was finalized in 2009, when the new competence center in Gothenburg offered its support to the founders. After a year of close collaboration between system developers, project managers and orthopedic surgeons, a beta version was launched on 1 January 2011 by the Department of Orthopedics and Trauma at Sahlgrenska University Hospital. Fractures of the tibia and humerus were entered during the trial period. The long bones, shoulder, pelvis and foot were included in April 2012, followed by the hand in October 2012 and the spine in February 2015.

The SFR is run by a national board that has members representing various parts of the country, orthopedic departments, specialties and academic disciplines. The board is supervised by a director who is responsible for maintaining and developing the register. The Swedish Orthopedic Trauma Society, a section of the Swedish Orthopedic Association, is the professional organization that provides support. Funding comes from the Western Healthcare Region and the Swedish Association of Local Authorities and Regions.

### Inclusion and exclusion criteria

The SFR collects data on all extremity, pelvic and spine fractures in adults who have been diagnosed or treated at affiliated departments. Data entry requires the patient to have a permanent Swedish personal identity number, be 16 years of age or older and have a fracture diagnosed on the basis of radiographs, Computed Tomography (CT scan) or Magnetic Resonance Imaging (MRI). Pediatric fractures are in the process of being added. Swetrau and other Swedish national quality registers focus on major trauma, whereas various other orthopedic registers specialize in non-skeletal injuries [12, 13].

### Technical description

The fully web based SFR is built on the new Stratum platform, designed specifically for health quality registers. Apart from the PROM questionnaires, no paper is used in the data entry process. The system provides users with input choices based on previously entered data, thereby speeding up the process and minimizing the risk of error, and permits the consecutive entry of new injuries, treatment and follow-up, including PROM. The vision from the outset has been to ensure user friendliness and an intuitive interface.

### The data entry process

**Fig. 1:**
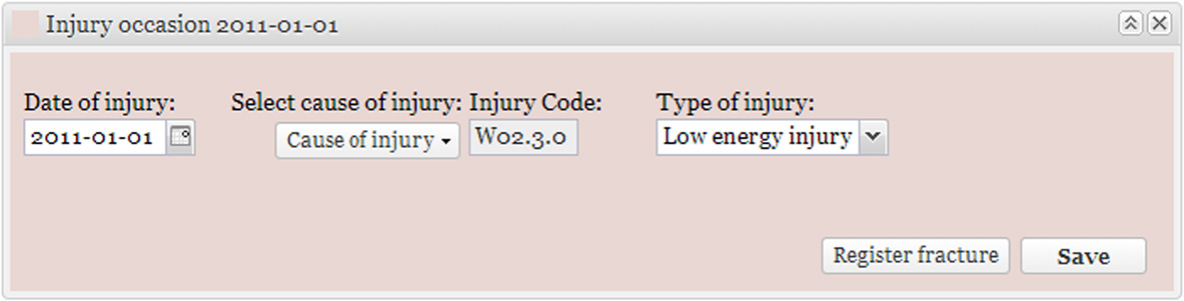
Registration of injury occasion. The date, cause, code and type (high-energy or low-energy) of injury are entered in the first panel. The mechanism, location and activity in which the patient was engaged when the injury occurred are chosen by means of drop-down menus that contain submenus for each specific variable, thus creating a V or W code in accordance with International Classification of Diseases Tenth Revision (ICD-10). Pathological, stress and spontaneous fractures are distinguished from traumatic fractures. The amount of energy that went into the injury is estimated on the basis of generally accepted criteria. Subsequent accidents can be added such that data relating to a particular fracture will always be associated with the correct injury and date

**Fig. 2:**
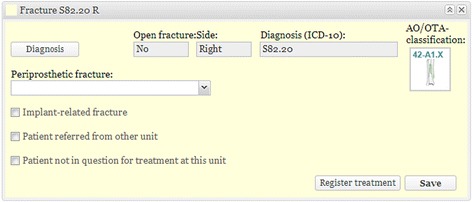
Registration of fracture. The second panel contains data relating to the fracture. The information is generated as soon as the physician chooses a location and side of the body, followed by a series of alternatives and answers to mandatory questions. The diagnosis is assigned ICD-10 codes, side of the body, information about whether the injury is open or closed and an AO/OTA class or other category. Boxes can be checked to indicate whether the fracture is related to a prosthesis or other implant. In the case of multiple fractures, a new panel is generated for each additional fracture. To make statistical analysis more reliable, boxes showing that the patient is being treated at another hospital are helpful in describing the chain of treatment

**Fig. 3:**
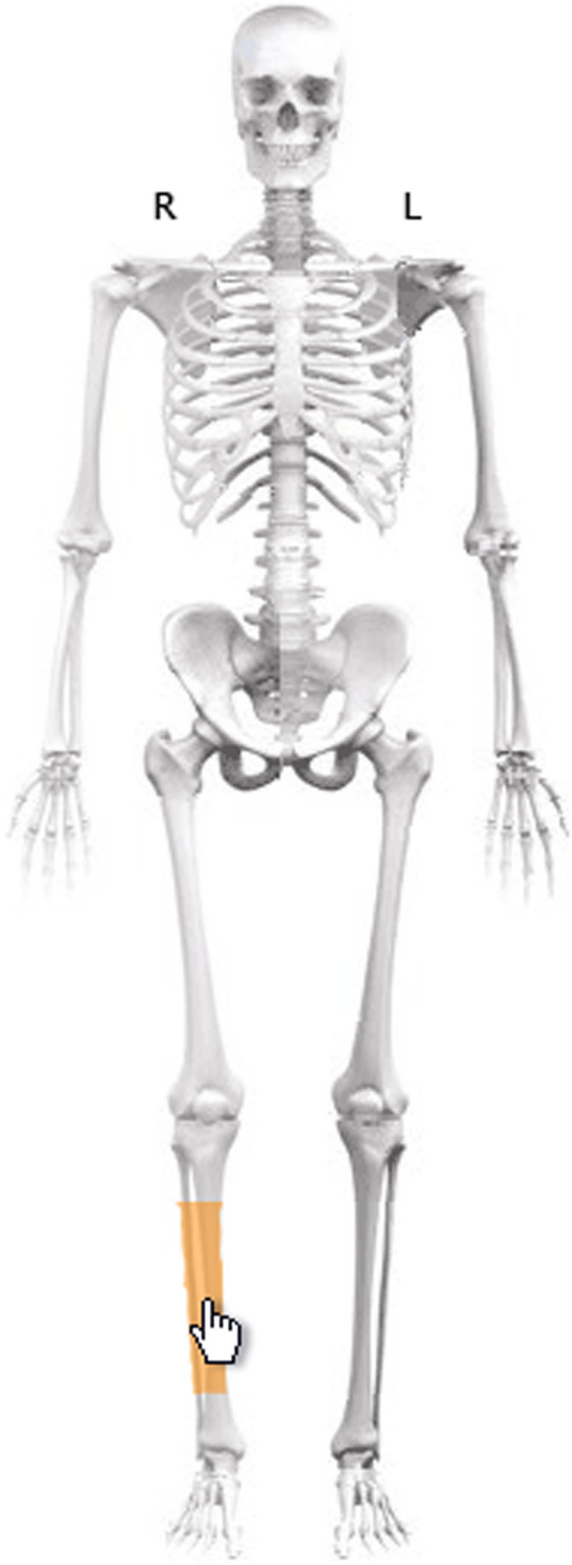
Classification of fracture. After selecting a segment on the skeleton, a classification window for the segment in question appears (Fig. 4)

**Fig. 4:**
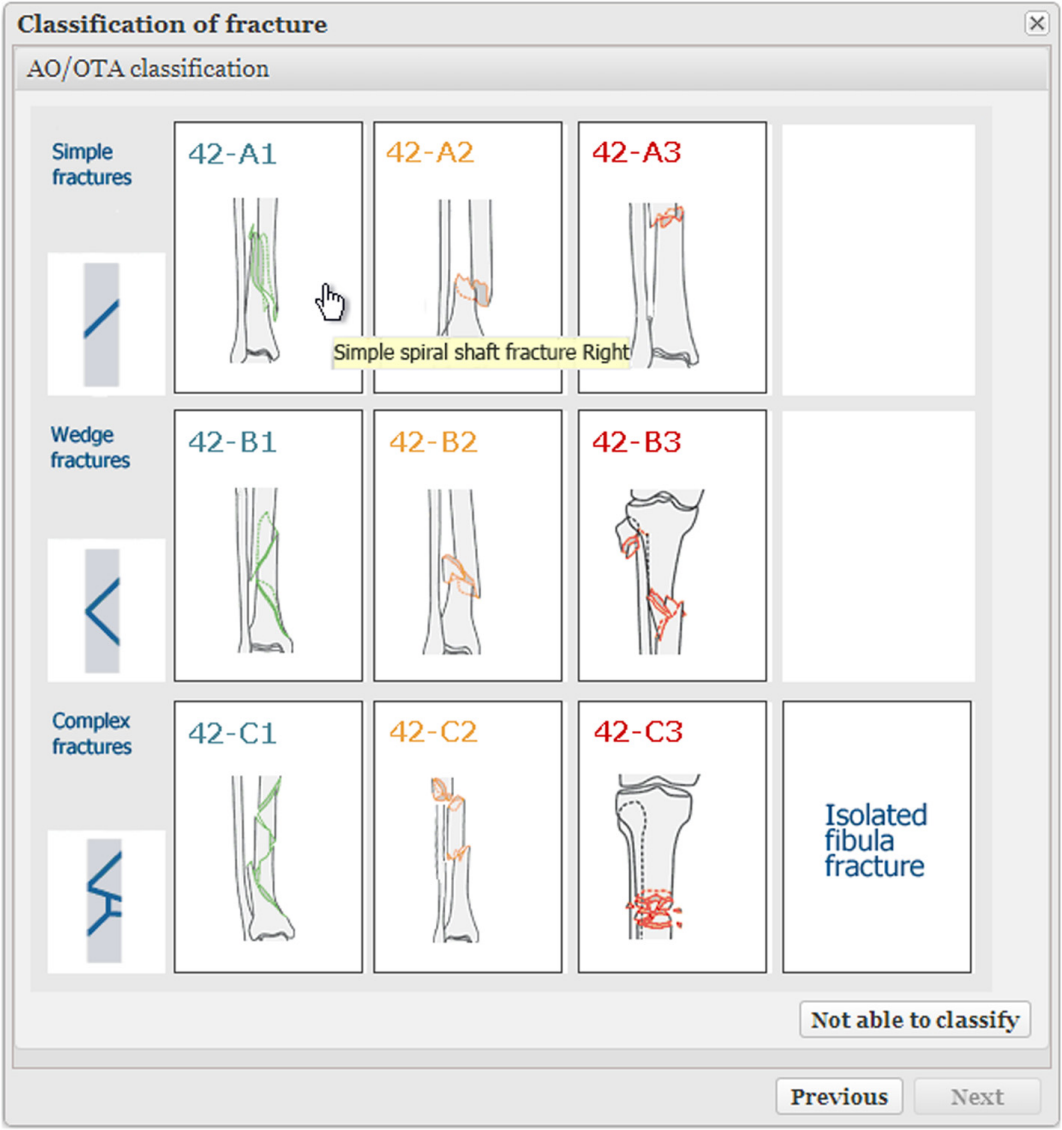
Classification of fracture. After the location and side of the body have been identified on the skeleton (Fig. 3), the classification window appears. Moving the cursor to a particular fracture category brings up a written description to supplement the drawing. Sample radiographs are also available. Optionally, final classification can be performed after surgery

**Fig. 5:**
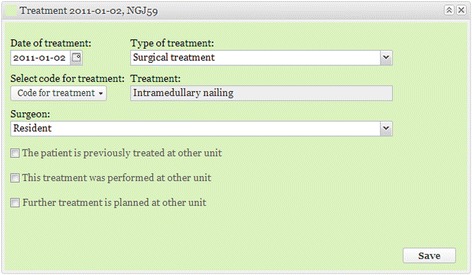
Registration of treatment. The third panel contains data on treatment. The date and type of treatment are chosen from drop-down menus. Only treatments possible for the particular fracture are shown. The registration of treatment includes information about the specific type of implant used, approaches and so on. Finally, the surgeon’s experience level is entered and boxes are checked to indicate whether additional surgery will be performed at another hospital. If the treatment plan is changed or a new procedure is performed, a second treatment panel opens to enable the entry of the additional data. All the color-coded panels can easily be minimized to provide an overview of the injuries and sequence of treatments

The data entry process consists of four different color-coded steps (Figs. 1, 2, 3, 4 and 5). The first three steps are performed by the physician, while the fourth step contains PROM. Data entry is performed by the attending physician, normally a specialist or resident in orthopedics and trauma, or by others who are on call at accident and emergency departments. They log in with a personal service identification card and a Personal Identification Number (PIN) code. The Swedish Personal Data Act mandates the two-step process. The patient’s personal identity number, an eight-digit date of birth and a unique four-digit control code, is then entered. The number is verified online with the Swedish Population Register and a new file is created if the number is correct.

The diagnosing physician enters the date and the cause of the injury and information about the fracture(s), generally classified in accordance with Arbeitsgemeinschaft für Osteosynthesefragen (AO)/Orthopaedic Trauma Association (OTA) [14, 15]. Classification is based on the available radiological information. A detailed description of the classification process appears later in this article*.* Treatment is entered once it has been performed. If the fracture is treated non-surgically, the physician on call enters this. If the fracture is treated surgically, the surgeon enters the data. If surgery is performed secondary to non-surgical treatment that has failed, the entire sequence of events is recorded. Scheduled secondary procedures are distinguished from reoperations, which are entered, along with the indications for which they were performed. The illustrations in Figs. 1, 2, 3, 4 and 5 show the data entry process. Data are stored under the responsibility of the County council and on computer servers run by the University of Gothenburg.

### Classifying the fracture

A fracture must be analyzed and described before it can be correctly treated. A classification system is essential to the success of a fracture register. Maurice E. Müller argues that classification is useful only if it considers the severity of the bone lesion and serves as a basis for treatment and for evaluating the results [16]. A classification system suitable for a fracture register should ideally be comprehensive, widely recognized, extensively employed, user friendly and valid. No current classification system meets all these criteria. However, AO/OTA is the best available option and we have chosen to use it whenever feasible and meaningful [14, 15]. It has been adapted to the demands of the register and to the online features that are particularly useful for pelvic, acetabular and forearm fractures. For example, pelvic fractures can be assigned an ICD code based on the individual fracture components indicated on a pelvic overview in the first step, with the AO/OTA code (instability pattern) generated in the next step. Similarly, proximal radius and ulna fractures are classified for each bone separately, ultimately linking them together. Acetabular fractures are classified in accordance with both AO/OTA and Letournel [17]. Hip fractures are classified in accordance with AO/OTA, but the descriptions associated with the diagrams refer to the Garden classification of cervical hip fractures [18]. In the same way, proximal humerus fractures are assigned an AO/OTA class, but the description parallels Neer’s terminology [19]. Clavicle fractures are classified in accordance with Robinson and scapula fractures are classified in accordance with Euler and Rüedi and Ideberg, whereas foot fractures are assigned a modified OTA code [20–22].

### Patient-reported outcome measures

Shortly after primary treatment at the hospital, two questionnaires are sent to the patient: the Euroqol 5 dimensions 3 level (EQ-5D-3L) and the Short Musculoskeletal Function Assessment (SMFA) [23–25]. The advantage of the EQ-5D is its widespread use by other registers and health-economic analyses, while, on the other hand, the SMFA permits a greater understanding of the patient’s musculoskeletal function. The questionnaires relate to health status and level of functioning the week before the fracture and are answered using recall technique. Responding patients are sent identical questionnaires one year later to assess their recovery. The completed questionnaires are electronically scanned centrally at the SFR. Their calculated indices are displayed in a fourth (grey) panel, without the responses to individual questions. Data are presented as a bother index and five different dysfunction indices. A few additional questions, such as smoking habits, have been added.

### The implementation process

Virtually all fractures in Sweden are treated at 55 publicly funded hospitals. All orthopedic and trauma departments at Swedish hospitals have been offered the opportunity to participate. The participating departments have received at least one visit by the register staff to aid the start-up. It normally takes 3–4 months for logistical issues to be ironed out and data entry to begin. As of 31 December 2014, 26 hospitals enter fracture data on a regular basis.

### Completeness

The register has a search function to identify incomplete data relating to the injury, fracture, treatment or PROM. Each department is free to incorporate methods of its own in order to ensure the most complete data possible. At the Sahlgrenska University Hospital structured searches in the digital medical records are being made. Each week the medical records are scanned for ICD-codes related to fractures. These search results are matched for entries in the SFR and the fractures that have not been registered in the SFR are secondarily registered. In this way all patients who have a fracture diagnosis in the medical chart are registered in the SFR. Some small departments that treat only a few fractures a day can simply check their daily radiograph demonstration.

### Retrieval of data from the register

A national health quality register has a better chance of succeeding and fulfilling its purpose if it is able to provide relevant real-time information to its users. Since the SFR relies on surgeons to find the time to enter data, the surgeon should preferably recognize the usefulness of the information in order to be fully motivated. Moreover, the medical directors need to appreciate the value of the information to promote the department’s participation. With this in mind, we have developed a dozen search functions that enable easy retrieval of data from the register. By combining different parameters, the user is able to obtain immediate, up-to-date statistics for both the particular department and the register as a whole. The data are presented in such a way that it is easy to save tables and diagrams. Another useful feature is the ability to create a search list for a particular type of fracture, gender, age group or treatment during a specified period at the user’s department. The register returns a list of personal identity numbers much more readily than most hospital databases are capable of doing. This function greatly facilitates internal quality control and targeted follow-up.

### Ethics

The Swedish Fracture Register is approved by the Swedish Data Inspection Board and operates in accordance with Swedish legislation i.e. the Swedish Personal Data Act and the Swedish Patient Data Act. All patients are informed that the registration takes place and that they have the right to decline, however according to Swedish legislation (the Swedish Patient Data Act) national quality registers do not need signed consent from the individual registered patient. The research conducted in the SFR was approved by the Central Ethical Review Board, Gothenburg. For research purposes, approval by the Regional Ethical Review Board has to be obtained prior to use of register data. If a single center intend to use data registered at their own center for local quality improvement, no such approval is needed. Centers are still encouraged to seek ethical approval as this will facilitate publishing of the results in peer reviewed scientific journals.

## Utility and discussion

The registration of fractures started in 2011, when fractures of the tibia and humerus began to be entered at Sahlgrenska University Hospital. The registration of fractures of the long bones, shoulder, pelvis and foot was included in April 2012, when other hospitals were also offered the chance to participate. Fractures to the hand were included in October 2012 and fractures of the spine in February 2015. The number of participating hospitals has risen from 7 in January 2013 to 26 in December 2014. They include university, general and district hospitals in various regions of the country. The total coverage of the hospitals affiliated to the SFR is approximately 5.5 million inhabitants (more than 50 % of the Swedish population).

Two annual reports have been published [26, 27]. An English translation of the second annual report for 2013 is available.

Tables 1, 2, 3 and 4 show the number of fractures, cumulative growth, gender and age distribution and distribution according to high- or low-energy injury in the SFR from the start in January 2011 until 30 September 2015. The total number of registered fractures as of 30 September 2015 is more than 103,000.

**Table 1:** Number of fractures and cumulative growth in number of fractures included in the SFR per quarter of the year (quarter 1, 2011-quarter 3, 2015)

Quarter of the year	Number of fractures	Cumulative number of fractures
2011 - Q1	225	225
2011 - Q2	185	410
2011 - Q3	233	643
2011 - Q4	211	854
2012 - Q1	296	1150
2012 - Q2	1227	2377
2012 - Q3	1440	3817
2012 - Q4	2225	6042
2013 - Q1	4741	10,783
2013 - Q2	5922	16,705
2013 - Q3	6909	23,614
2013 - Q4	7529	31,143
2014 - Q1	8356	39,499
2014 - Q2	8202	47,701
2014 - Q3	8802	56,503
2014 - Q4	11,005	67,508
2015 - Q1	13,273	80,781
2015 - Q2	11,551	92,332
2015 - Q3	11,573	103,905

**Table 2:** Gender distribution of patients in the SFR (1 January 2011–30 September 2015)

Women	Men	Total
55285	38,851	94,136
59 %	41 %

**Table 3:** Age distribution of patients in the SFR (1 January 2011–30 September 2015)

Age (years)	Number of patients	Percent
16–20	4964	5
21–30	9312	10
31–40	7138	8
41–50	9391	10
51–60	12,738	13
61–70	15,714	17
71–80	14,136	15
81–90	15,544	17
91–100	5093	5
>100	106	0.1
Total	94,136

**Table 4:** Number of injuries in the SFR distributed according to high- or low-energy injury (1 January 2011–30 September 2015)

Cause of injury	Number of injuries	Percent
High energy	8373	9
Low energy	83,061	87
Unknown	4190	4
Total	95,624	

During the four years and three-quarters that the SFR has been active, more than 103,000 fractures at 26 different orthopedic departments have been entered. Together, these 26 orthopedic departments cover more than half the Swedish population. Nevertheless, many issues remain to be discussed and resolved. The greatest possible completeness (entry of a satisfactory percentage of the fractures treated at each department) is the most important goal, followed by high coverage (participation rate among the departments that treat fractures). The objective is for participating departments to report every fracture that they treat. This goal is ambitious and even the most efficient register is unable to reflect fracture incidence with 100 % accuracy. However, there are several ways of ensuring a satisfactory level of completeness and it is up to the various departments to choose a sustainable approach to enter the fractures they treat. One way to identify missing entries is to create a digital tool (such as a search function) to find fracture patients in the medical records and match them with register entries as is done at the Sahlgrenska University Hospital. Studies on completeness are currently being conducted. Attaining the goal of nationwide coverage is a daunting challenge, given that entry in the SFR is not compulsory. The success of the well-known Swedish registers for hip and knee arthroplasties is based on registers run by the orthopedic profession and not being mandatory. For these registers the implementation process to achieve full coverage among the orthopedic departments in Sweden took about 10 years. The variables in a fracture register are probably even more complex than in hip or knee arthroplasty registers. It might therefore take a long time to achieve full coverage in a fracture register.

As measures are adopted to achieve these goals, the register continues to evolve. The Swedish Spine Association has decided to start entering spinal fractures, both spontaneous/low-energy fractures in the elderly and traumatic/high-energy fractures, while phasing out the recording of these fractures in its Swespine register. This process began in February 2015. Pediatric fractures are in the process of being incorporated. In the pediatric segment of the register, interest will focus on long bone fractures. The assignment of a fracture to an adult or pediatric category will be based not on age but on the maturity of the skeleton (whether the epiphyseal lines of the affected bone are open or closed).

The validity of data is of the utmost importance if they are to be used for scientific and quality improvement purposes. The SFR and its users share the responsibility for ensuring that data are entered in an appropriate manner. On-going validation efforts are required. Studies are being conducted to control for fracture classification accuracy, completeness with respect to national statistics, accuracy in the entry of reoperations and polytrauma data in acute settings. The ability to draw meaningful conclusions from PROM questionnaires requires either a high response rate or evidence that respondents do not significantly differ from non-respondents. Studies are being conducted in this area as well.

The SFR is unique since it is population based, covers fractures of all types, regardless of treatment, and collects both surgeon- and patient-reported outcome measures. Its vision for the future is to supply researchers and health-care providers with population-based data that add to the body of knowledge on the treatment of fractures. Many orthopedic surgeons and organizations are involved in a valiant effort to collect this data day by day. When combined with appropriate scientific analysis, the SFR will be able to serve as a springboard for improving health care and raising the standards that patients demand and expect. The SFR will be able to present both the results of fracture treatment and valuable epidemiological data.

The SFR is able to deliver data to other national quality registers or to the patients’ medical records but the Swedish legislation restricts the exchange of information between registers. However, with legal and ethical consent, research that combines data from different national registers can be performed. Comorbidities and medications are currently not registered in the SFR but, with legal and ethical consent, analyses of co-morbidity and potential confounders are possible by obtaining information from other national registers. In addition to providing an enormous prospectively collected database on every aspect of fracture treatment, we expect the SFR to become the starting point for recruiting patients to specific research and healthcare projects. For example the SFR is currently used for screening patients for osteoporosis.

It will take a number of years for the SFR to achieve a satisfactory level of completeness and coverage. Most orthopedic registers, both in Sweden and abroad, are fairly simple by comparison, but they, too, generally need several years before their goals can be fully realized.

## Conclusions

Three years after the release of the current version of the SFR, 26 orthopedic and trauma departments have started to enter the fractures they treat either surgically or otherwise. The success of initial implementation makes it clear that satisfactory compliance with the aims of the register is possible and that surgeons can find the time to perform the required data entry. In the future the SFR will be able to present both results of fracture treatment and valuable epidemiological data.

## Availability and requirements

The Swedish fracture register website is found at www.frakturregistret.se

Log in process and restriction to use is described in the 'Construction and content' section of this paper.

## Abbreviations

AO: Arbeitsgemeinschaft für Osteosynthesefragen
CT: Computed Tomography
EQ-5D-3L: Euroqol 5 dimensions 3 level
ICD-10: International Classification of Diseases Tenth Revision
MRI: Magnetic Resonance Imaging
OTA: Orthopaedic Trauma Association
PIN: Personal Identification Number
PROM: patient-reported outcome measures
SFR: Swedish Fracture Register
SMFA: Short Musculoskeletal Function Assessment
